# Closing the gap: a roadmap to single‐cell regulatory genomics

**DOI:** 10.15252/msb.20209497

**Published:** 2020-05-19

**Authors:** Julie Carnesecchi, Ingrid Lohmann

**Affiliations:** ^1^ Department of Developmental Biology Centre for Organismal Studies (COS) Heidelberg Heidelberg University Heidelberg Germany

**Keywords:** Chromatin, Epigenetics, Genomics & Functional Genomics, Computational Biology, Development & Differentiation

## Abstract

Studying the spatiotemporal control of gene regulatory networks at the single‐cell level is still a challenge, yet it is key to understanding the mechanisms driving cellular identity. In their recent study, Aerts and colleagues (González‐Blas *et al*, 2020) develop a new strategy to spatially map and integrate single‐cell transcriptome and epigenome profiles in the *Drosophila* eye‐antennal disc and to deduce in each cell precise enhancer‐to‐gene activity relationships. This opens a new era in the transcriptional regulation field, as it allows extracting from each of the thousands of cells forming a tissue the critical features driving their identity, from enhancer sequences to transcription factors to gene regulatory networks.

## Graphical abstract

Analysing gene regulatory networks at the single‐cell level has remained challenging. In their recent work, Aerts and colleagues (González‐Blas *et al*, 2020) develop a strategy to spatially integrate single‐cell transcriptome and epigenome data and infer enhancer‐gene activity relationships in each cell.

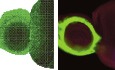

Cellular identity is specified by the combination of genes active within a cell, controlled by gene regulatory networks (GRNs). These cell‐specific networks are turned on by transcription factors (TFs), which interact with specific sequences in accessible chromatin regions, promoters and enhancers. Although seemingly straightforward, it is so far highly challenging to comprehensively and simultaneously retrieve all the information on TF‐controlled regulatory networks from individual cells forming a tissue, organ or organism without losing spatial information. Yet, this is critical to go beyond a mere description of cell identities based on gene expression profiles and to understand the mechanisms controlling the formation of individual cell types with distinct identities in the organismal context.

Single‐cell sequencing approaches are instrumental in achieving this goal. However, while single‐cell RNA‐sequencing (scRNA‐seq) is extensively used to profile GRNs (Karaiskos *et al*, 2017; Fiers *et al*, 2018), and single‐cell epigenomics methods allow profiling the chromatin landscape and active regulatory regions in individual cells (Cao *et al*, 2018; Cusanovich *et al*, 2018), it is currently difficult to determine both the transcriptome and epigenome of the same cell. Moreover, although computational algorithms have been developed to integrate independent single‐cell datasets (Stuart *et al*, 2019; Welch *et al*, 2019), they are still limited with regard to data source and biological information. Thus, a comprehensive characterization of gene regulation in individual cells of complex tissues is so far missing despite being key for our understanding of cell identity control *in vivo*.

In their recent study, González‐Blas *et al* (2020) integrate single‐cell transcriptomics and epigenomics data and deduce genome‐wide precise enhancer‐to‐gene relationships in single cells of a tissue. Their work closes an important gap in the transcriptional regulation field, and their multi‐dimensional approach (Fig 1) was key for achieving this. Most importantly, they selected an extremely well‐suited biological system for spatial modelling of gene regulation, a 2D tissue composed of complex yet spatially restricted cell populations, the well‐characterized *Drosophila* eye‐antennal disc. Next, they developed a suite of tools and algorithms to spatially map single‐cell transcriptome and epigenome profiles to their putative position of origin by building a virtual representation of the 2D tissue that mimics the organization and structure of the eye‐antennal disc. They then used this information to computationally link enhancers and genes within each virtual cell and validated the enhancer‐to‐gene relationships using reporter fly lines from the Janelia FlyLight project (Jory *et al*, 2012). Analysing this single‐cell enhancer‐to‐gene resource in detail revealed that gene expression control is even more complex than anticipated, as 80% of the genes within a cell are regulated by shadow enhancers with an average of 22 enhancers linked to one gene, and to even more when the gene encodes a TF. González‐Blas *et al* then went on to define within the thousands of enhancers active in a cell those sequences critical for enhancer function by identifying naturally occurring cis‐regulatory variation in inbred lines using chromatin accessibility quantitative traits loci (caQTLs). By correlating these bulk data with known TF motifs and their single‐cell resource, they were able to infer the precise cell type in which variations in TF binding sequences are relevant, and thereby identified Prospero as a neuronal differentiation factor acting in photoreceptor cells via a GGG motif that would have been missed by standard motif search engines.

**Figure 1:**
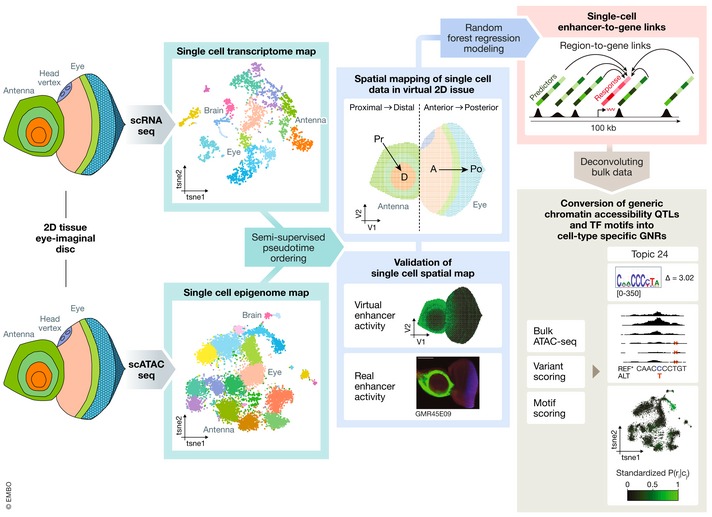
A roadmap to single‐cell regulatory genomics González‐Blas *et al* generated independent single‐cell RNA‐seq and single‐cell ATAC‐seq atlases of the *Drosophila* eye‐antennal disc and spatially integrated them using a virtual latent space that mimics the organization of the 2D tissue. Predicted enhancers were validated using the Janelia Flylight collection of enhancer‐reporter lines, which confirmed the coupling of chromatin accessibility and enhancer activity. Using random forest regression models, enhancer‐to‐gene relationships were calculated in each cell, which revealed that gene regulation is highly redundant and each gene is controlled by multiple enhancers. The single‐cell information was finally used to deconvolute cell‐type specific effects of bulk derived chromatin accessibility data, which identified new motifs and TFs active in specific cell types of the eye‐imaginal disc (Several elements shown in this Figure have been reused from González‐Blas *et al*, 2020, including the virtual enhancer activity map, the confocal image showing the enhancer activity in the eye‐antennal disc and the example of the chromatin accessibility QTL, Topic 24).

The study by González‐Blas *et al* is a major break‐through, as their multi‐dimensional approach for the first time enables a comprehensive analysis of *bona fide* GRNs in single cells, from TFs interacting with specific binding sequences in enhancers to the expression of target genes. This is a huge step towards single‐cell regulatory genomics and thus is of invaluable importance for the community. One surprising result of this work is that a large fraction, about 50%, of the enhancer‐to‐gene links show negative correlation, indicating that accessible chromatin regions not only correspond to promoters, insulators or primed/active enhancers but also to repressed enhancers. These regions very likely control the repression of alternative fate genes, which is known to be equally important for the cell‐type specification. Nevertheless, this finding has to be taken into account particularly when attempting to generate spatial maps of a tissue based only on single‐cell epigenomics data, as it indicates that open chromatin does not by default mean active regulatory regions. Another intriguing result of this study is that TFs are regulated by the highest number of redundant enhancers, which supports previous notions that the control of higher‐level regulators needs to be much more elaborate than the one of terminal effectors. Importantly, the computational method integrating single‐cell omics data proposed by González‐Blas *et al* is the starting point for assembling GRNs within organized tissues. It will be interesting to see whether it can be applied to 3D organoid models to decrypt the spatial and regulatory information at the level of cell‐to‐cell communication in pathological cell models characterized by heterogeneous populations. Another exciting use of this algorithm is the dynamic visualization of GRNs at the temporal scale. It would be remarkable to use it for visualizing gene expression during the specification of one cell type and combining the transcriptional dynamic with the usage of regulatory enhancer elements throughout tissue development and differentiation. One can be truly eager to follow the progress of single‐cell omics data coverage and its computational integration for understanding cell and tissue biology at the single‐cell level.

## References

[msb209497-bib-0001] Cao J , Cusanovich DA , Ramani V , Aghamirzaie D , Pliner HA , Hill AJ , Daza RM , McFaline‐Figueroa JL , Packer JS , Christiansen L *et al* (2018) Joint profiling of chromatin accessibility and gene expression in thousands of single cells. Science 361: 1380–1385 30166440 10.1126/science.aau0730 PMC6571013

[msb209497-bib-0002] Cusanovich DA , Reddington JP , Garfield DA , Daza RM , Aghamirzaie D , Marco‐Ferreres R , Pliner HA , Christiansen L , Qiu X , Steemers FJ *et al* (2018) The cis‐regulatory dynamics of embryonic development at single‐cell resolution. Nature 555: 538–542 29539636 10.1038/nature25981 PMC5866720

[msb209497-bib-0003] Fiers MWEJ , Minnoye L , Aibar S , Bravo González‐Blas C , Kalender Atak Z , Aerts S (2018) Mapping gene regulatory networks from single‐cell omics data. Brief Funct Genom 17: 246–254 10.1093/bfgp/elx046 PMC6063279 29342231

[msb209497-bib-0004] González‐Blas CB , Quan XJ , Duran‐Romaña R , askiran I , Koldere D , Davie K , Christiaens V , Makhzami S , Hulselmans G , de Waegeneer M *et al* (2020) Identification of genomic enhancers through spatial integration of single‐cell transcriptomics and epigenomics. Mol Syst Biol 16: e9438 10.15252/msb.20209438 PMC7237818 32431014

[msb209497-bib-0005] Jory A , Estella C , Giorgianni MW , Slattery M , Laverty TR , Rubin GM , Mann RS (2012) A survey of 6,300 genomic fragments for cis‐regulatory activity in the imaginal discs of *Drosophila melanogaster* . Cell Rep 2: 1014–1024 23063361 10.1016/j.celrep.2012.09.010 PMC3483442

[msb209497-bib-0006] Karaiskos N , Wahle P , Alles J , Boltengagen A , Ayoub S , Kipar C , Kocks C , Rajewsky N , Zinzen RP (2017) The *Drosophila* embryo at single‐cell transcriptome resolution. Science 358: 194–199 28860209 10.1126/science.aan3235

[msb209497-bib-0008] Stuart T , Butler A , Hoffman P , Hafemeister C , Papalexi E , Mauck WM , Hao Y , Stoeckius M , Smibert P , Satija R (2019) Comprehensive integration of single‐cell data. Cell 177: 1888–1902.e21 31178118 10.1016/j.cell.2019.05.031 PMC6687398

[msb209497-bib-0010] Welch JD , Kozareva V , Ferreira A , Vanderburg C , Martin C , Macosko EZ (2019) Single‐Cell multi‐omic integration compares and contrasts features of brain cell identity. Cell 177: 1873–1887.e17 31178122 10.1016/j.cell.2019.05.006 PMC6716797

